# A study of organizational versus individual needs related to recruitment, deployment and promotion of doctors working in the government health system in Odisha state, India

**DOI:** 10.1186/s12960-016-0103-1

**Published:** 2016-02-24

**Authors:** Shridhar Kadam, Srinivas Nallala, Sanjay Zodpey, Sanghamitra Pati, Mohammad Akhtar Hussain, Abhimanyu Singh Chauhan, Sovesh Das, Tim Martineau

**Affiliations:** Indian Institute of Public Health, Bhubaneswar, 2nd & 3rd Floor, JSS Software Technology Park, E1/1, Infocity Road, Patia, Bhubaneswar, 751024 India; Public Health Foundation of India, Delhi NCR, Plot No. 47, Sector 44, Institutional Area, Gurgaon, 122002 India; Division of Epidemiology and Biostatistics, School of Population Health, The University of Queensland, Herston Road, Herston, Brisbane, QLD 4006 Australia; Department of International Public Health, Liverpool School of Tropical Medicine, Pembroke Place, Liverpool, L3 5QA UK

**Keywords:** Recruitment, Deployment, Policy, Doctors, Odisha, India

## Abstract

**Background:**

An effective health workforce is essential for achieving health-related new Sustainable Development Goals. Odisha, one of the states in India with low health indicators, faces challenges in recruiting and retaining health staff in the public sector, especially doctors. Recruitment, deployment and career progression play an important role in attracting and retaining doctors. We examined the policies on recruitment, deployment and promotion for doctors in the state and how these policies were perceived to be implemented.

**Methods:**

We undertook document review and four key informant interviews with senior state-level officials to delineate the policies for recruitment, deployment and promotion. We conducted 90 in-depth interviews, 86 with doctors from six districts and four at the state level to explore the perceptions of doctors about these policies.

**Results:**

Despite the efforts by the Government of Odisha through regular recruitments, a quarter of the posts of doctors was vacant across all institutional levels in the state. The majority of doctors interviewed were unaware of existing government rules for placement, transfer and promotion. In addition, there were no explicit rules followed in placement and transfer. More than half (57%) of the doctors interviewed from well-accessible areas had never worked in the identified hard-to-reach areas in spite of having regulatory and incentive mechanisms. The average length of service before the first promotion was 26 (±3.5) years. The doctors expressed satisfaction with the recruitment process. They stated concerns over delayed first promotion, non-transparent deployment policies and ineffective incentive system. Almost all doctors suggested having time-bound and transparent policies.

**Conclusions:**

Adequate and appropriate deployment of doctors is a challenge for the government as it has to align the individual aspirations of employees with organizational needs. Explicit rules for human resource management coupled with transparency in implementation can improve governance and build trust among doctors which would encourage them to work in the public sector.

## Background

Health workers in sufficient numbers, appropriately placed, adequately supported and motivated are the backbone of an effective, equitable and efficient public health care system [1]. The World Health Organization has emphasized this as a critical component to achieve health-related Millennium Development Goals [2] and reinforced its importance for achieving the new Sustainable Development Goals. However, many low- and middle-income countries still face challenges in recruiting and retaining health staff in the public sector [3, 4]. Further, inappropriate posting and transfer of health workers compromise the health system goal of providing equitable and efficient health care [5].

In India, several national policy and review documents have highlighted an insufficient number of doctors in the government health system throughout the country [6, 7]. This has been a matter of concern for the Government of India and various state governments [8]. Odisha, one of the Indian states with low health indicators, is still grappling with a shortage of doctors with a 24% vacancy at various levels in most of the districts [9]. The number of doctors per 10 000 population in Odisha is three times lower than that of other states like Goa and Kerala [1]. Recognizing the need for attracting and retaining more doctors in the public health system, the Government of Odisha has initiated efforts through the provision of incentives and recruitment drives. Yet, the availability of doctors in rural and hard-to-reach areas is still below the desired levels especially at the primary health centres (PHCs), the peripheral units of the government health system [10].

According to McGregor, the greatest challenge in human resource management (HRM) is to integrate the needs of the organization with the individual needs of its members [11]. Employers may develop deployment (placement and transfer) rules to meet the needs of the organization; nevertheless, employees will make their choices considering health facility infrastructure, living conditions, career opportunities and professional satisfaction [12]. For instance, non-transparent deployment policies, delayed recruitment, inadequate incentives and limited opportunities for career development have negative effects on attracting and retaining doctors in public health system in India [13–15].

Therefore, it is necessary to align recruitment, deployment and promotion policies with the needs of the organization, while being effective in attracting, retaining and motivating the staff. It is equally important to develop guidelines for the implementation of such policies, mechanisms for incentives and accountability [16] and consider employee perspectives while making decisions [17].

In this paper, we examined the policies on recruitment, deployment and promotion for doctors in the state of Odisha (the systems as should be—de jure) and how these policies were perceived to be implemented by the stakeholders (to identify the practice—de facto). Drawing on data from document review and in-depth interviews with doctors and key informants, we analysed how these rules and their implementation are aligned with organizational needs and explored their congruence with the needs of the doctors affected by them.

## Methods

### Study setting

The 30 administrative districts of Odisha, with a total population of 41 947 358, are categorized into 11 KBKs (Koraput, Bolangir and Kalahandi) which comprise 25% of the population and 19 non-KBKs (75% of population) based on socio-economic indicators. KBK districts represent the southern part of Odisha, comprising a mostly tribal population with poor living conditions and an underdeveloped economy [18]. All rural areas in KBK districts are designated as tribal.

Each district has a three-tier health care delivery structure: primary health centres (PHCs) at the village level (for 30 000 population), community health centre (CHC) at the block level (for 100 000 population) and district hospital (DH) and sub-divisional hospitals (SDHs) at the district level with an average population of 1.3 million. PHCs and CHCs provide primary health care while specialist services are delivered at the SDH and DH. The data collection and analysis was carried out from July 2012 to March 2013.

### Data sources

The data sources comprise document review, key informant interviews and in-depth interviews with doctors. Through document review and key informant interviews, we delineated the existing policies for recruitment, deployment (initial posting and transfers) and promotion. We conducted in-depth interviews with doctors to examine the perceived level of compliance with these policies.

**Fig. 1:**
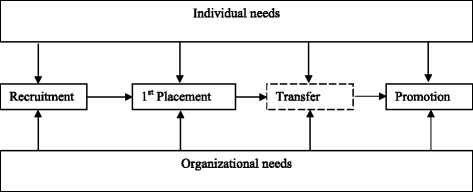
Conceptual framework for analysing factors related to recruitment, deployment and promotion of government doctors in Odisha. Source: developed by authors based on McGregor [11]

Based on McGregor’s challenge for HRM [11], we developed a framework (see Fig. 1) to capture information on the organizational needs and individual (doctor’s) aspirations related to recruitment, first placement, transfer and promotion. The box for transfer is shown as dotted because it is not a mandatory event as compared to others.

### Document review

We reviewed policies on recruitment, deployment and promotion for doctors within both national and international contexts. We analysed available reports and documents of the Government of Odisha related to recruitment, deployment and promotion such as the State Service Codes (1959) and the State Service Manual (2004) [19]. We collected these documents from government sources such as the High Court of Odisha, Gazette notification of Odisha and General Administration Department. In addition, relevant peer-reviewed and grey literature on recruitment, deployment and promotion policies and practices was included for review.

### Key informant interview (KII)

We interviewed four senior administrators with responsibilities for HRM from the state level to confirm the information on rules and policies extracted from official documents and government websites. We elicited their views on the implementation of existing rules and policies regarding recruitment, deployment and promotion of government doctors and sought their suggestions for reforming these rules and policies for better implementation. We used a semi-structured topic guide for interview.

### In-depth interview with doctors

The interviews covered four areas: (1) the respondent’s socio-demographic characteristics; (2) respondents’ job history starting from the date of joining government service on ad hoc, contractual or permanent basis and the location, type of institution, duration, designation, their job responsibility of each posting and reason for their transfer to the next posting whether personal or job related; (3) respondents’ knowledge about existing policy and rules related to recruitment, placement, transfer, incentives and grievance redressal; and (4) their level of satisfaction with the existing policy and expectations. We used a pre-tested semi-structured interview schedule to collect this information. This tool was also used to collect quantitative information such as demographic profile, length of services and number of transfers.

In order to have an adequate representation from both the KBK as well as non-KBK districts, we proportionately selected six districts—two out of 11 KBKs and four from 19 non-KBK districts. We interviewed 90 doctors (86 from six districts and four working at state level). We used multi-stage stratified sampling and selected 12, 23, 15, 20, 16 and four doctors working at the primary health centre (PHC), community health centre (CHC), sub-divisional hospital (SDH), district hospital (DH), district health office (DHO) and state health office (SHO), respectively. As there were less number of female doctors working within the government health system, we interviewed female doctors in situations where both male and female were available.

The principal investigator (SK) and four co-investigators (SN, MAH, ASC and SD) conducted all interviews. We took permission from the state and district health authorities prior to the data collection process. Each interview ranged from 45 to 60 min and was digitally recorded.

### Data management and analysis

The interviews were transcribed verbatim and translated to English. The interview data were given unique IDs and stored in a password-protected folder. Two members of the research team who are fluent in both English and the local vernacular language did the translation. The English transcripts were then imported to NVivo (QRS NVivo Version 8.0.180.0 SP1) for data coding and analysis. We used “thematic framework approach” to analyse the qualitative data [20]. This approach comprises data familiarization, coding, theme identification, charting and interpretation.

The quantitative data from the semi-structured interviews was entered and analysed in Microsoft Excel, and descriptive statistics was computed.

### Ethical considerations

Ethical clearance was obtained from the Institutional Ethics Committee of Public Health Foundation of India, New Delhi. The study was also approved by the Research Committee, Department of Health and Family Welfare, Government of Odisha. Informed consent was obtained from all the study participants. All steps were taken to maintain confidentiality and anonymity of the respondents.

### Limitations of the study

As our sample size was small to get in-depth responses from the respondent, we do not claim the views expressed are representative of doctors in the state of Odisha or other states in India. Further, we have included perceptions of only one type of stakeholders, i.e. doctors on implementation of policies. Undertaking multi-stakeholder perspectives might yield a more comprehensive picture on the practice of policies.

## Results

Table 1 summarizes the findings on the policies related to recruitment, deployment and promotion, their implementation and views and suggestions of doctors interviewed.

**Table 1:** Summary of findings

Policy	Implementation and views of the doctors interviewed	Suggestions by doctors interviewed
Recruitment		
Explicit rules for recruitment done by OPSC for permanent positions. Open to all medical doctors with a maximum age limit of 32 years. DOHFW can do direct recruitment for temporary and ad hoc positions. DOHFW at the state level is responsible for all recruitments.	Recruitment was done as per the set rules. OPSC exams were conducted annually. Two thirds of doctors interviewed were aware of the recruitment processes. However, scoring and ranking system in OPSC exams was not clear to them. Doctors interviewed preferred to join government service only after selection through OPSC.	Continue current recruitment policy through OPSC with more clarity on scoring and ranking system. Recruitment on ad hoc basis should be stopped.
Deployment		
It is mandatory for each government doctor to serve at least 3 years in the KBK districts. No clearly delineated guideline for placement or transfer of doctors across primary, secondary and tertiary care facilities. Any doctor can be transferred across the state after 3 years of service in a single location. DOHFW at state level is responsible for deployment of recruited doctors.	Out of 56 doctors interviewed from non-KBK districts, 32 (57%) had never worked in KBK districts. Two thirds of doctors interviewed remained in the same region (KBK or non-KBK) where they had started their service. There were 3.0, 2.0 and 2.2 average transfers in the first, second and third decade of their service, respectively. This was less than the stipulated policy of minimum 3 years of service to be eligible for transfer. Placements and transfers of doctors were done centrally. Majority of the doctors interviewed were not satisfied with the current practice of placements and transfer.	Deployment based on clear and fixed criteria. Designation of hard-to-reach areas should not be restricted only to KBK districts but also to other areas which are hard to reach. District authorities should be allowed to deploy doctors based on local needs. A time-bound and transparent transfer policy based on rotation.
Incentives		
Monthly hardship allowance of 8 000 Indian Rupees (around 20% of new entrant’s salary) for rural and 4 000 Indian Rupees for urban areas in KBK districts. Monthly allowance of 3 000 Indian Rupees for specialist doctors.	All the doctors interviewed from the KBK district were receiving hardship allowance. However, they perceived it as insufficient and ineffective. Limited awareness among non-KBK district doctors interviewed on incentives given in KBK districts.	Hardship allowance in proportion with degree of remoteness and linked with performance.
Promotion:		
State-level Departmental Promotion Committee that is independent of DOHFW is constituted to look after the promotions of doctors. The criteria for promotion is based on vacancy, OPSC ranking, length of service and performance appraisal report. Doctors are to be designated as specialist after acquiring postgraduate degree. 50% of postgraduate seats in government medical colleges are reserved for in-service doctors.	Around half the doctors interviewed were aware about the rules and process of promotion. Delayed first promotion with quick promotions in later career. Majority of junior doctors interviewed were not satisfied with the slow pace of the promotion. The average time interval in designating a doctor as a specialist after getting specialist degree was 7.5 (±6.3) years. Out of 90 doctors interviewed, 49 had done postgraduation of which 31 had done through in-service quota.	The criteria for promotion should include numbers of years’ service in rural area and performance in addition to length of service and OPSC ranking.

Of the 90 doctors interviewed, 73 (81%) were male and 17 (19%) were female. The median age of respondents was 52 years. The average length of service of the study participants was 21.4 (±9.7) years (Table 2). The average length of service in tribal, rural and urban areas was 7.4, 10.4 and 12.5 years, respectively. Of the 86 doctors interviewed from six districts, 56 (65%) were from non-KBK districts and 30 (35%) from KBK districts.

**Table 2:** Posting and length of service of study participants working in remote (KBK districts) and non-remote (non-KBK districts)

Sl. no.	Non-KBK district interviewees	*n* = 56	KBK district interviewees	*n* = 30
1.1	Started service in KBK	17 (30%)	Started service in KBK	22 (73%)
1.2	Started service in non-KBK	39 (70%)	Started service in non-KBK	8 (27%)
2.1	Served some time in KBK	24 (43%)	Served some time in non-KBK	15 (50%)
2.2	Never served in KBK	32 (57%)	Never served in non-KBK	15 (50%)
3.1	Average length of service	21.4 years	Average length of service	20.4 years
3.2	Average service duration in KBK	2.5 years	Average service duration in KBK	13.7 years
3.3	Average service duration in non-KBK	18.9 years	Average service duration in non-KBK	6.7 years

The following sections describe the current policies (de jure) on the recruitment, deployment and promotion of doctors working in the government health system.

### Recruitment policy

The Odisha Public Service Commission (OPSC) recruits doctors on behalf of the Odisha Department of Health and Family Welfare (DOHFW) for permanent posts through written examination followed by personal interviews [21]. In addition, the DOHFW recruits doctors on an ad hoc basis in temporary positions as and when required. Temporary or ad hoc positions are for a limited period of time, and renewal depends on the requirement. Such doctors need to apply for the OPSC exams to be placed on regular service. The maximum age for entry into service through OPSC is 32 years.

### Deployment policy

Deployment (including placement and transfer) of recruited doctors within the state is done centrally by the DOHFW [22]. The policy implies that a doctor can be placed or transferred anywhere in the state and there are no explicit criteria for placement and transfer. It is mandatory for each government doctor to serve at least 3 years in the KBK region. A doctor can be transferred after 3 years of service in a single place based on vacancy and/or individual request [19].

All the 11 KBK districts are designated as hard-to-reach areas. Doctors working in these districts are given a monthly hardship allowance of 8000^1^ Indian Rupees for rural areas (about 20% of their salary) and 4000 in urban areas (about 10% of their salary). However, those who are involved only in administrative work do not receive this allowance. Further, there is a provision of additional 10% marks per year of service to a maximum of 30% in the postgraduate entrance examination for these doctors.

After acquiring postgraduation degree, doctors get designated as a “specialist” and are posted at the CHC level and above. They receive an incentive of 3000 Indian Rupees per month once designated as a specialist. Fifty percent of the postgraduate (PG) seats in state-owned medical colleges are currently reserved for in-service doctors. There are opportunities for doctors to undergo short-term trainings and diploma courses in both clinical and public health disciplines.

### Promotion policy

The Departmental Promotion Committee (DPC) at the state level deals with the promotions of doctors. It is an independent committee chaired by a member from the Department of General Administration and two to four members from the DOHFW [21]. Promotions are done based on vacancy, OPSC ranking, length of service and performance appraisal report. The performance appraisal has to be done annually and forwarded by the chief district medical officer (CDMO) to the Department of Health and Family Welfare and Department of General Administration [23]. Performance appraisal reports for the previous 5 years are considered when a doctor is due for promotion. Responding to the concerns raised by the association of doctors in 2009 about the lack of promotion prospects, the Government of Odisha restructured the doctor’s cadre with a change in the entry level position from Class II to Junior Class I and increased the senior level positions with higher salary grades so as to create more opportunities for promotion. The revised grades of promotion are ranked from Junior Class I, Senior Class I, Joint Director Level II, Joint Director Level I, Additional Director, Director and Special Secretary. Again, in 2011, the Government of Odisha increased the number of positions of Additional Director. All the Junior Class I positions are tenable at the PHC level or at the CHC, SDH or DH level as general duty medical officer. All the positions of Senior Class I and above can be tenable at the CHC, district or state level. Director and Special Secretary can only be posted at the state level.

### Grievance redressal policy

There is a fixed day every week (Monday) for grievance redressal at the state level. Doctors who have any grievance related to deployment, promotion and any other concern regarding their job can approach the head of the DOHFW (through proper channel, i.e. CDMO). The head of the DOHFW listens to the grievances of doctors and take appropriate measures to redress them.

The following sections describe the perceived practice of policy (de facto) on recruitment, deployment, promotion and grievance redressal, as reported by the medical officers.

### Recruitment practice

The key informants said that the OPSC examinations were being held annually since 2006, with additional rounds in 2006, 2009 and 2012. In 2013, all the selected doctors were placed in KBK districts and designated tribal areas of non-KBK districts. Generalist doctors were posted at the PHC level while doctors with selected postgraduate disciplines were placed against vacancies of specialist positions at the CHC, SDH and DH.

Most of the doctors working at the field level said that the existing recruitment process is acceptable. However, their major concerns were the irregularity of the OPSC examinations and lack of clarity on the recruitment process, particularly on the ranking and scoring system.

Recruitment is through OPSC, which should be held regularly. I had to wait for about three years for the OPSC exams.(specialist doctor working at DH)

Two thirds of the doctors interviewed were aware of the official recruitment process for both permanent and temporary posts. The doctors who were not aware of the recruitment process were older (median age of 53 years—range 28 to 59) and had more years of service (median years of 27 years of service—range 1 to 32).

### Deployment practice

Analysis of job histories of doctors interviewed shows that around half of them were placed at the PHC while the remaining were placed at the CHC, district or other hospitals in urban areas on their first posting. The majority of males were posted at the PHC whereas females were mainly placed in urban areas.

The majority of doctors interviewed reported that there is no specific policy for placement, and it is usually done based on vacancy rather than qualification or length of service. Even a doctor with PG qualification could be placed at the PHC level that is not designated for specialist health care. This was perceived by doctors as an underutilization of skills. Furthermore, a candidate’s choice of posting is not taken into account while allocating placement.

Most of the doctors interviewed stated that the first posting should be in the KBK region as per the government’s stipulated policy, but their perception was that this is seldom practised. There had been instances where doctors were posted directly in an urban health facility at the district or state level or even at the medical colleges on their first placement and not at the PHC. Job histories also revealed that out of eight doctors recruited in last 3 years, four were posted in KBK and four in non-KBK.

Some doctors thought that the placement system is not transparent and depends on the individual’s ability to influence the system to get their preferred posting. Once posted, representation for relocation (to a more preferable location) may be considered, but mostly, the decision by the government is final. The doctors working as district level managers said that all placements are done centrally, and they do not have the authority to modify the placements as per local needs.

There are no such rules [for placement]. It’s done at state level. If there were rules for placement, after completion of my post-graduation, I should have been placed at district head quarter hospital but this was not the case (district level programme manager)

There should be a definite structure. New recruits should be in PHC level for five years, then in CHC for five years and SDH or DH for next ten years and subsequently posted to directorate (specialist doctor working at DH)

The doctors interviewed from the KBK districts said that other than the hardship allowance no incentive is being given to them. The doctors from the non-KBK region were not getting any incentives and were unclear about the incentives for working in KBK districts. Some of them did not know whether such incentives were given while some others, though aware about the incentives, did not know the exact amount.

I don’t know about any specific incentive scheme for KBK districts (medical officer at CHC in non-KBK district)

I have heard about the extra incentives given in KBK districts but don’t know the exact amount (surgery specialist from district hospital in non-KBK district)

A few senior medical officers were against the incentive system. According to them, such incentives neither attract more doctors to government service nor guarantee physical presence at their place of posting despite receiving incentives.

No need for incentives, everybody should serve in KBK areas for a certain period (specialist doctor from sub-divisional hospital)

Incentives may not ensure physical presence of doctor in rural or remote PHCs. You can find doctors taking incentive and managing without being there at all (district-level programme manager)

The job histories of doctors interviewed revealed that the number of transfers during their service tenure varied from none to 18 with an average of 5.1. Further, the average number of transfers in the first, second and third decade of their service was 3.0, 2.0 and 2.2, respectively. No difference was observed in the number of transfers between male and female doctors as well as those working in *KBK* or *non-KBK* districts.

The average length of service of doctors interviewed working in the KBK and non-KBK was almost equal, that is, 20.4 and 21.4 years, respectively. Two thirds of doctors interviewed remained in the same region (either KBK or non-KBK) where they had started the service. Half of the those interviewed from KBK districts have worked in non-KBK districts with an average length of 6.7 years, whereas 57% of doctors interviewed from the non-KBK district never worked in KBK districts and 43% worked with an average length of 2.5 years (Table 2).

Two thirds of the doctors interviewed stated that there are no clear rules for transfers. They said that transfers are mostly based on vacancy of a position and requirement of personnel at a given place. A few were of the opinion that the transfers sometimes are used as punitive measures. Few also told that knowing someone in the hierarchy or good contacts with politicians or informal payments play a role in getting a transfer or avoiding it.

The majority of doctors said that there is no fixed tenure followed for posting in KBK or non-KBK districts. They further said that as the KBK districts are difficult areas and less popular, if someone gets transferred to this region, there is limited opportunity of getting back to non-KBK districts.

Once posted in KBK, there is no escape (specialist doctor working at DH)

### Promotion practice

The average length of service of the doctors interviewed before their first promotion (from Junior Class I to Senior Class I) was 26 (±3.5) years. The average time interval for second- and third-level promotions was 1.4 and 0.96 years, respectively. Out of a total of 90 doctors interviewed, 49 had postgraduate qualification. Of these, 31 doctors had done postgraduation as an in-service candidate while 18 had done postgraduation prior to joining the service. The average time interval in designating a doctor as a specialist after getting PG degree was 7.5 (±6.3) years.

The majority of the doctors interviewed were unhappy with the delay in their first promotion. While recently promoted senior officers expressed satisfaction about their rapid promotion in the previous 2 years, they were of the opinion that it should have happened much earlier. Doctors with postgraduate qualification expressed their displeasure that they were not promoted as specialists even though they knew that there were vacancies.

I have been working in this set up for 20 years but not been given any promotion. The pay scale is low. There is no scope for academic growth and job satisfaction (medical officer working at PHC).

It is a delayed mechanism. It is on the basis of vacancy and not in years of service rendered. I have been only promoted after 25 years. At my stage, I should be JD-II, but I don’t know when it is going to happen (specialist doctor working at DH)

Around half of the doctors interviewed were aware of promotion procedures. Most of them were of the view that getting a promotion in the early phase of one’s career plays a vital role in motivating and retaining personnel in service while delayed promotion leads to frustration and dissatisfaction.

### Grievance redressal practice

The majority of the doctors reported that the grievance redressal is regularly held on each Monday. However, they expressed concerns such as (1) Time allotted for each doctor to express the concerns is inadequate; (2) Long travel as the grievance redressal is held only at the state level and it is difficult to leave the work station; (3) It is a cumbersome process to access the office of the DOHFW; and (4) Not all grievances are redressed satisfactorily and influence or payments are expected by the lower level staff.

### Suggestions by doctors for improving recruitment, deployment and promotion system

On recruitment policy, the overall opinion of doctors was positive. However, they suggested a few changes like conducting OPSC recruitment annually and to make the process more transparent especially the evaluation of candidates. Ad hoc or temporary recruitment should be discouraged because this brings in complacency in conducting regular OPSC and there is no preferential selection of those already in ad hoc or temporary service.

The majority of the doctors suggested that deployment should be based on clear and fixed criteria taking into consideration their educational qualification, work experience, choice of posting and native place. They also suggested that areas that are difficult in non-KBK districts should also be considered by the government while the designation of “hard to reach” and therefore incentives should be given to staff working in all hard-to-reach areas and not restricted to KBK locations only. Another suggestion was to provide additional allowance which should be based on the degree of remoteness of the health facility. A time-bound and transparent transfer policy was found to be the most frequently expressed need. This entails that a doctor who has joined the government service is informed beforehand the period for which they are expected to serve in each location. Further, the total duration of service in a rural or urban area should be delineated from the very beginning. Rural service should be made compulsory for everyone with periodic transfers between rural and urban. Preference in transfers should be given to those with service experience in rural or hard-to-reach areas.

Most of the doctors suggested that instead of vacancy-based promotion, a system for time-bound promotion could be adopted. A few were of the opinion that promotion should take cognizance of factors such as service duration, rural service, education qualification and performance in addition to OPSC ranking.

## Discussion

Our study examined current policies on recruitment, deployment and promotion for doctors in the state and explored their implementation in practice. Using McGregor’s challenge for HRM [11], we critically analysed the issues related to these policies and practices from both individual and organizational perspectives.

### Recruitment

Despite the efforts of the Government of Odisha to recruit doctors through regular OPSCs, there are about 24% vacancies of doctors in the state. To mitigate this challenge, the government also recruits doctors on an ad hoc basis so as to bring more doctors into the systems between the time intervals of recruitment through the OPSC. However, the doctors prefer to join through the OPSC than ad hoc because of the benefits associated with OPSC selection like job security and promotion avenues [8]. A study conducted in Viet Nam observed that a permanent job with a stable source of income was an important motivational factor for health staff to join government service [24].

In our study, out of 49 doctors with postgraduate qualification, 31 had done postgraduation as an in-service candidate while 18 had done postgraduation prior to joining the service. This suggests that though the distribution of postgraduate seats is 50:50 for in-service and fresh candidates, there are fewer fresh postgraduate doctors joining or continuing the government service and they might be joining the more lucrative private sector. It is argued that the urban-based, specialized care and hospital-centred model of medical education can lead to a situation where physicians with specialization are more likely to work in the private sector and in urban areas [25]. Furthermore, most of the undergraduate medical students prefer to do postgraduation prior to joining service [26]. Therefore, the current policy for 50% postgraduate seat allocation for in-service doctors may be helpful in retaining in-service doctors though may not be that much effective to attract fresh candidates. More so, many of these candidates while investing their time in preparation for PG entrance examinations may cross the age limit of 32 years set by OPSC and would not be able to enter the government service. Raising OPSC age limit for entry into government service can increase the pool of potential candidates to join government service.

Increased production of doctors, regular OPSC and additional rounds of ad hoc appointments may be necessary but might not be sufficient to attract and recruit more doctors in the public health system. Despite regular OPSC, there is still 24% vacancy which necessitates tracing eventually how many doctors join and continue government service after OPSC selection and placement. Since in-service doctors after their postgraduation tend to remain within the government system, creating greater opportunities for higher education for in-service doctors might improve attraction and retention.

### Deployment

The appropriate distribution of doctors to ensure equity in access to health care is a concern as well as priority for every government. In this regard, the Government of Odisha has made it compulsory for doctors to serve at least 3 years in KBK districts and other designated hard-to-reach areas. On the other hand, doctors will always prefer posting in urban areas for better amenities and greater professional growth [5, 25]. Although it is mandatory for all doctors to serve in KBK districts for a minimum of 3 years, contrastingly, job histories revealed that majority of the doctors interviewed from non-KBK districts had never worked in the KBK region. Further, for those who have worked, the tenure was 2.5 years which is less than the stipulated policy. In this case, the organizational objective of providing doctors for hard-to-reach areas is not met and the reason could be that the managers are not pursuing the organizational interest and might be benefitting someway by favouring the individual interests of the employees [5].

The majority of doctors interviewed remained in the same category (KBK or non-KBK) of district where they had started their service. This suggests that the first posting could be critical in determining their service trajectory. The doctors perceive that once they are posted in KBK districts it would be difficult to get their choice of posting afterwards. A few believe that influence and informal payments can help in getting their choice of posting. Given these perceptions, doctors will try to manoeuvre their first posting so as to get their preferred location. If unsuccessful, either they would not join or might join for a brief period and discontinue later or may take extended leave and remain continually absent from the workplace [5].

We believe that every employee would try to achieve their individual interests such as posting in urban areas and expect a fair treatment from the organization. If they perceive that the system is not fair to them in the implementation of rules and policies, some may try to get round the rules and others may leave the organization. Therefore, it is the responsibility of the government to balance the organizational needs and individual aspirations through transparent rules and uniform implementation. As seen in our study, even if there are explicit rules, implementation was not uniform owing to individual interests dominating over organizations’ firmness. A recent attempt was made by the government in 2013 to ensure that all the newly recruited doctors were posted in KBK districts and designated tribal areas of non-KBK districts. However, guaranteeing that doctors actually report for duty and then stay in these posts is a challenge for the government [5].

Interestingly, we found that even though there is a skewed deployment of female doctors interviewed in urban areas on their first posting, subsequently, there was no difference in the number of transfers between male and female. There could be two possible explanations for this—(a) female doctors’ options for urban posting might have been considered favourably by the government and (b) females might be showing willingness to join only if given the urban posting. However, equal number of transfers among male and female suggest that the system is not always open to manipulation by a particular group.

A number of authors claim that job satisfaction plays key role in employee retention [24, 27–30]. Therefore, posting a specialist at the PHC level is not only underutilization of skill from the organizational point of view but also frustrating for the employees [31]. Similarly, posting a new recruit generalist doctor directly to a higher level institution is unfair to senior generalists who are continuing to serve at PHCs in a rural area without getting the opportunity to move to an urban area. Having a specific policy or rules for posting doctors across levels of health facilities is equally crucial for creating a fair mechanism of deployment.

The majority of doctors interviewed are not satisfied with the current practice of transfer. Organizations may make the transfers primarily for ensuring equitable distribution. Another objective may be to bring fairness in the system so as to rotate people from rural to urban areas after a fixed tenure. However, the employees may want transfer for getting their choice of posting. The actual posting and transfer can be a negotiated outcome of preferences and objectives of the organization and the individual employee [5]. However, there might not be always a match between the objectives of the organization and those of the individuals. If the employees feel that the system is not transparent and fair, they would not trust the system. Declaring vacancies of health facilities and inviting choices by doctors at the time of transfer could bring in more transparency and trust in the system while taking care of organizational needs.

The main purpose of the hardship allowance provided in KBK districts is to attract and retain doctors in this region. Our study revealed that these incentives are neither lucrative nor effective in ensuring attraction and retention of doctors in these districts. In this context, it is necessary to understand that the managers and health staff may perceive incentives differently; what managers consider as lucrative may not appear so for the health staff [28]. Further, for any incentive system to be effective, it is important that employees are informed about it. We found that respondents from non-KBK districts are not well aware of the incentives given in KBK districts. This poses a question as to how can the system attract its employees to rural and remote areas if they do not even know about the incentive and do not find the incentive attractive enough.

### Promotion

We found that for all the doctors interviewed, the first promotion is delayed followed by rapid promotions at the tail end of their career. One of the explanations for this could be that there is little turnover at the higher level; thus, few vacancies are created. Similarly, with low production and inconsistent recruitment of doctors, vacancies at the primary care level remain high. This poses a dilemma for the government as it has pressure from the public to post more doctors at the periphery and simultaneously they require people at the higher level for administration and management. However, once a doctor is promoted, they cannot be posted at the PHC. Hence, to address the deficit of doctors at PHCs, the government might have to restrict promotion. This results in stagnation on the career ladder for doctors which, according to respondents in this study, is de-motivating. More so, potential entrants may not be attracted to join the state government service given such restricted career growth opportunities.

## Conclusions

Recruitment and deployment are essential human resource management functions to ensure adequate availability of staff and its equitable distribution in order to achieve greater access and better health care services. However, we found that it is not always easy to align the individual aspirations of the employees with organizational needs regarding these functions, especially where there is a staff shortage. Doctors want an urban posting as well as promotion after certain years of service whereas the government continues to deploy them at the periphery to meet the health service demands of the population. Deployment of doctors in the periphery for long duration without any promotion does lead to frustration. On the other hand, promoting doctors would result in vacancies in health facilities in the periphery. Therefore, it is necessary to consider individual aspirations and organizational needs while designing policies for recruitment, deployment and promotion.

Furthermore, when the systems are pliable and policies are ambiguous, there is always a scope for its distortion and manipulation by both employees and managers. In Odisha, there is a government policy for doctors on compulsory service in hard-to-reach areas; however, this policy is not implemented in its full sense as there is no transparency in placements and transfers. In such an environment, some will distort the system for their own benefit while others will feel the system is being unfair to them. Improving transparency and knowledge about rules is essential; however, until the misuse of these rules by managers is addressed, the necessary trust will not be developed, and it will remain challenging to influence the behaviour of medical officers to support equitable deployment.

We should also recognize that the interplay of individual and organizational needs occurs in a broader environment of social context [25]. The government as an employer should ensure that the organizational goals are clear and policies are aligned to motivate individuals to contribute to the system [32]. Systematically analysed and strategically developed long-term plans are essential to deal with the balance between the needs of the individual and those of the organization. This could help meet the new Sustainable Development Goals in India and other health workforce-constrained countries.

## Abbreviations

CDMO: chief district medical officer
CHC: community health centre
DH: district hospital
DOHFW: Department of Health and Family Welfare
DPC: Departmental Promotion Committee
HRM: human resource management
JD: joint director
KBK: Koraput, Bolangir and Kalahandi
OPSC: Odisha Public Service Commission
PG: postgraduate
PHCs: primary health centres
SDH: sub-divisional hospitals

## Footnotes

1 1 US dollar = 55 Indian Rupees at the time of study
